# Lifestyle Variation among the Elderly: Do Nutritional Knowledge and Diet Quality Differ When the Other Lifestyle Components Are Similar?

**DOI:** 10.3390/life13102057

**Published:** 2023-10-14

**Authors:** Marzena Jeżewska-Zychowicz

**Affiliations:** Department of Food Market and Consumer Research, Institute of Human Nutrition Sciences, Warsaw University of Life Sciences (SGGW-WULS), Nowoursynowska 159C, 02-776 Warsaw, Poland; marzena_jezewska_zychowicz@sggw.edu.pl; Tel.: +48-22-5937131

**Keywords:** the elderly, lifestyle, diet, physical activity, sleep duration, smoking, alcohol drinking

## Abstract

Older people’s health is strongly determined by their lifestyle, and its deterioration is a cause for concern and calls for effective efforts to slow down the process. The aim of the study was to determine the relationship between diet quality and other non-food lifestyle elements. The data were collected in 2017 through a cross-sectional survey among 271 Polish elder citizens. A K-means cluster analysis was applied to separate homogeneous groups by lifestyle components (without diet) and a logistic regression was used to analyze the relationships between identified clusters and sociodemographic characteristics, nutritional knowledge and diet quality. Three homogenous clusters were identified, i.e., individuals with moderate physical activity and recommended sleep duration (pro-healthy), with low physical activity (low PA), and with short sleep (short sleep). Gender, age, education, place of residence, BMI, and health-promoting diet index (pHDI) did not differentiate adherence to clusters. The probability of being in the pro-healthy cluster increased with nutritional knowledge and declaring the same or better health status compared to peers, while it decreased when above-average financial status was reported. The obtained results importantly extend the previous findings by showing that the individual elements of lifestyle should be also perceived in the context of others. Further research focused on lifestyle as a whole might considerably support the implementation of multifaceted lifestyle interventions.

## 1. Introduction

The aging of societies has precipitated in recent decades due to an increase in life expectancy and a decrease in the fertility rate. The fact that people live longer has brought along the desire to remain healthy in later life while the lifestyles of older people are deteriorating. At the same time, there is substantial evidence from epidemiological studies among older adults that individual lifestyle factors, including physical activity, alcohol consumption, smoking, and body weight, are strongly associated with their health and functioning [1,2,3,4]. Leading a healthy lifestyle is paired with a slower decline in physical, psychological, cognitive, and social functioning with aging [4].

Nevertheless, research shows a reduced prevalence of healthy lifestyle factors in adults including older adults with the exception of smoking [5]. The overall lifestyle deterioration of older adults may negatively impact their functioning and therefore hamper successful aging [4]. However, the results are not consistent across groups with specific sociodemographic characteristics [6,7]. The residents of rural areas demonstrated significantly more healthy lifestyles in terms of physical activity and diet than the residents of cities [7]. Moreover, women are more likely than men to lead healthy lifestyles by means of following more balanced and healthy diets [8], and consuming fewer stimulants such as tobacco and alcohol [1]. The differences were also found in terms of education, with generally lower education being associated with less healthy behaviors [9,10]. However, there are also studies that show no such differences, for example, Zanjani et al. [11] have not confirmed any significant correlation between the level of education and the lifestyle of the elderly. This demonstrated diversity, as well as constantly changing external factors, requires monitoring and response in order to counteract possible deterioration in the lifestyles of the elderly and hence in their health.

Research to date has mostly looked at single lifestyle components or at most two such components at once, e.g., diet and physical activity [12,13,14], physical activity and sleep [15,16,17], or diet and sleep [18,19]. There are still not enough observational and intervention studies taking into account more lifestyle elements at the same time to ensure more comprehensive results [20,21,22]. Instead, the available results indicate that all lifestyle elements are bilaterally correlated and that their impact on health is a comprehensive effect [1,22,23]. Yet, specific combinations of lifestyle risk behaviors may be more harmful than individual behaviors [24].

All lifestyle components including diet, physical activity, smoking, alcohol drinking, and sleep are modifiable under the influence of various stimuli both dependent and independent of the individual, e.g., actions taken by the individual to improve health [21] or changes forced by a pandemic [25]. The deterioration of older people’s lifestyles calls for effective education programs and interventions aimed at beneficial changes in the lifestyles of older people. Existing programs have focused more on diet [26,27] than on other lifestyle components, so increasing knowledge of the relationship between diet and other lifestyle components in older people will fill in the missing knowledge, while also finding practical applications [28]. Hence, the aim of the study was to determine the relationship between diet quality, nutritional knowledge and other non-food lifestyle elements as determinants of a homogeneous lifestyle. It was hypothesized that a more adequate diet and higher nutritional knowledge characterized people who displayed a more health-promoting lifestyle including physical activity, sleep duration, smoking, and alcohol consumption. In addition, sociodemographic characteristics, BMI, and subjective health assessment were used for profiling the identified homogenous lifestyles.

## 2. Materials and Methods

### 2.1. Ethical Approval

The study was approved by the Ethics Committee of the Faculty of Human Nutrition and Consumer Science, Warsaw University of Life Sciences, Poland, on 19 June 2017 (Resolution No. 22/2017). The study was conducted according to the guidelines laid down in the Declaration of Helsinki. After explaining the purpose of the survey, the interviewer asked the respondent for consent to participate in it.

### 2.2. Study Design

The data were collected through a cross-sectional quantitative survey using the computer assisted personal interviewing (CAPI) technique by the research agency KANTAR TNS in November–December 2017. The sample was a random, stratified representation of the total adult population of Poland. Details regarding recruitment protocol and methodology were previously reported [29]. The group whose responses were analyzed in this study included 271 persons aged 60 and over who were pensioners with no professional activity.

### 2.3. Questionnaire

The frequency of consumption of ten food groups was assessed with the beliefs and eating habits questionnaire (KomPAN) [30], which was validated for Polish adults [31]. The participants reported the habitual frequency of eating food in the three months preceding the survey using one of the answers: 1—less than once a month or never; 2—1–3 times a month; 3—once a week; 4—a few times a week; 5—once a day; and 6—a few times a day. During the data analysis, the answers were converted to reflect the daily frequency of intake, ranging from 0—less than once a month or never; 0.06—1–3 times a month; 0.14—once a week; 0.5—a few times a week; 1—once a day; and 2—a few times a day [32]. Pro-healthy dietary index (pHDI) was calculated based on 10 food groups with a potentially positive effect on health [32].

Self-reported physical activity was recorded in the questionnaire on a scale ranging from 1—‘low’ and 2—‘moderate’ to 3 –‘high’. The description of the scale was presented separately for the physical activity in leisure and work/school time. For leisure time, ‘low’ means ‘sedentary lifestyle, watching TV, reading the press, books, light housework, taking a walk for 1–2 h a week’; ‘moderate’—‘walks, cycling, gymnastics, gardening or other light physical activity performed for 2–3 h a week ‘; and ‘high’—‘cycling, running, working on a plot or garden, and other sports activities requiring physical effort, taking up more than 3 h a week’. ‘Low’ activity at work/school time was described as ‘over 70% of the time in a sitting position’, ‘moderate’ as ‘approximately 50% of the time in a sitting position and about 50% of the time moving’, and ‘high’ as ‘about 70% of the time in motion or doing physical work associated with a lot of effort’ [30].

Nutritional knowledge was evaluated using 25 statements with three response categories: 1—I do not agree, 2—I agree, and 3—I do not have an opinion. The answers were recoded based on the agreement or disagreement with the statement for correctness of the respondent’s answer (1—correct answer, 2—incorrect answer). When the nutritional knowledge was assessed, one point was awarded for a correct answer (1), whereas no points were awarded for an incorrect answer (2), and for not having an opinion (3) [30]. The total nutrition knowledge score was calculated as a sum of points (the range: 0–25 points). The higher the score the higher the nutrition knowledge level was.

Sleep duration was recorded separately for weekdays and weekend days on a three-point scale ranging from 1 to 6 h or less a night (short sleep); 2–7 or 8 h a night (optimal sleep); and 3–9 or more hours a night (long sleep).

Self-reported health was assessed using a three-point scale: 1—worse than peers; 2—same as peers; 3—better than peers. Self-reported financial status was assessed using a five-point scale: 1 —We live very modestly—we do not have enough money for basic needs; 2—We live modestly—we have to be very careful with our daily budget; 3—We live normally—we have enough money for our daily needs, but we need to budget for bigger purchases; 4—We are relatively wealthy—we have enough money for our needs without particular budgeting; 5—We are very wealthy—we can afford some luxury. Before analysis, responses were grouped according to the scheme: 1 and 2—below average; 3—average; 4 and 5—above average.

The questionnaire collected information about sociodemographic characteristics of the sample as well, including gender, age, education, and place of residence. Data about weight and height were self-reported by the participants. Body mass index (BMI) was calculated and interpreted according to the criteria of the World Health Organization [33].

### 2.4. Statistical Analysis

Descriptive statistics were used to present the characteristics of the study sample.

The normality of variables was checked by the Kolmogorov–Smirnov test. Comparison analysis between the subsamples was performed using the Kruskal–Wallis test (continuous variable) and χ^2^ test (categorical variables). A *p*-value lower than 0.05 was considered significant for all tests.

A K-means cluster analysis was applied using variables that described lifestyle, i.e., self-reported physical activity, sleep duration, smoking cigarettes, and alcohol drinking, to separate homogeneous groups (clusters). The sociodemographic variables, BMI, nutritional knowledge, and pHDI were used in cluster profiling.

Logistic regression analysis was used to verify associations between sociodemographic variables, BMI, nutritional knowledge, and pHDI (independent variables), and the clusters (dependent variables). Odds ratios (ORs) represented the probability of adherence to the clusters. The reference groups (OR = 1.00) were those representing other clusters. Wald’s test was used to assess the significance of ORs.

All analyses were performed with IBM Statistics SPSS, version 27.0 (IBM Corp., Armonk, NY, USA).

## 3. Results

### 3.1. Characteristics of the Study Sample

The majority of respondents were women (68.6%). More respondents (55.7%) lived in urban areas compared to rural areas (44.3%). More than two-thirds of the group had primary education (32.1%) and vocational education (36.5%). The majority of the respondents (62.3%) rated their health as the same as their peers. Only 9.6% of respondents were of normal weight, while the others were overweight (62.7%) or obese (27.7%) (Table 1). The average age was 70.8 years (SD = 6.69).

### 3.2. Socio-Demographic and Lifestyle Characteristics of the Clusters

The clusters identified based on to the lifestyle characteristics are presented in Table 2. Smoking cigarettes and frequency of alcohol consumption did not differ between the identified clusters. The cluster with low physical activity (low PA) was the most numerous, followed by the pro-healthy cluster i.e., moderate physical activity and adequate sleep duration (7–8 h). About one-fifth of the respondents represented the short sleep cluster.

There were no differences between the clusters in terms of socio-demographic characteristics (gender, age, education, and place of residence) with the exception of self-reported financial status. The pro-healthy cluster was represented by a larger number of people with “average” financial status compared to the other clusters. Similarly to the low PA cluster, there were also more people with “below average” financial status than in the short sleep cluster. In the low PA cluster, as well as in the short sleep cluster, there were more people declaring an above-average financial situation than in the pro-healthy cluster.

The majority of the respondents who perceived their health as better than their peers were in the pro-healthy cluster, and the smallest number were in the low PA cluster. The fewest people perceiving their health as worse than their peers were in the pro-healthy cluster, and the most of them were in the short sleep cluster. The biggest number of respondents describing their health as the same as their peers were in the pro-healthy and low PA clusters, and the smallest in the short sleep cluster. Cluster adherence was not differentiated when taking into account BMI (Table 2). People in the pro-healthy cluster were characterized by a higher nutritional knowledge compared to the other two clusters, while at the same time, there were no differences in the nutritional knowledge of people belonging to the latter clusters. There were no differences amongst clusters after adjusting for pHDI (Table 3).

The probability of belonging to the pro-healthy cluster increased with nutritional knowledge. Each subsequent point of nutritional knowledge increased the adherence to this cluster by 18% (Table 4).

People in this cluster were almost five times more likely to describe their health status as “same as peers” and more than 7.5 times more likely to consider it to be “better than peers” compared to other clusters. However, the probability of adherence to this cluster decreased by 70% for respondents who declared above average financial status. People in the low PA cluster were less likely to declare better health status than peers compared to other clusters. People in the short sleep cluster were less likely to display higher nutritional knowledge. Each subsequent point of nutritional knowledge decreased the adherence to this cluster by 9% (Table 4).

## 4. Discussion

Lifestyle components such as self-reported physical activity, length of sleep, alcohol consumption frequency, and smoking were used to identify homogeneous lifestyles in the study group. The analysis identified homogeneous groups after taking into consideration physical activity and sleep duration, while the other components had no differential effect. It has been shown that sleep and physical activity interplay physiologically and psychologically through multiple complex interactions [34]. In general, physical activity is beneficial for sleep, however it can be affected by a variety of factors, including but not limited to gender, age, fitness level or exercise characteristics. Conversely, sleep disturbances can decrease exercise activity and increase the risk of exercise-related injuries [35]. Previous studies have reported that elderly people aged ≥65 suffer from a variety of sleep problems [36] including both short and long sleep, which may also confirm the validity of inclusion of sleep in the cluster analysis.

The frequency of alcohol consumption and cigarette smoking did not contribute to identifying homogeneous groups. Instead, the results of available studies suggest that there is a positive relationship between physical activity and alcohol consumption [37,38]. Moreover, a higher physical activity was characteristic of alcohol consumers with a higher level of education, low TV viewing, and non-tobacco smokers [38]. Previous studies also showed that there is a negative correlation between cigarette smoking and the quality of physical activity [39], moreover, physical inactivity was associated with smoking in old adults [40,41]. Bilateral relationships between these components were not confirmed in a more comprehensive cluster analysis. It can be hypothesized that both alcohol consumption and cigarette smoking show a stronger relationship with individual lifestyle elements, while this effect diminishes when multiple elements are considered simultaneously. The lack of a differential effect of alcohol consumption may result from the measure used, that is, the frequency of alcohol consumption, without assessing the amount consumed. It is also worth noting that diet, with which other lifestyle elements are most often confirmed to be associated, was excluded from the cluster analysis and treated as a variable describing the identified clusters. The results encourage the continuation of research focused on lifestyle as a whole, which will make it possible to assess the importance of individual elements perceived in the context of others.

Three homogenous groups were identified in the study sample, i.e., individuals with moderate physical activity and recommended sleep duration (pro-healthy cluster), with low physical activity (low PA cluster), and with short sleep duration (short sleep cluster). It is known that these two components of the lifestyle may play a key role in attenuating the effects of aging on cognitive health [17,42]. In our study, respondents characterized by adequate sleep duration included both those with low physical activity (low PA cluster) and those with moderate physical activity (pro-healthy cluster), which may result from the fact that regular exercise has smaller beneficial effects on sleep duration than sleep quality [43,44]. The results of some studies suggest that elderly individuals who reported short sleep duration were less likely to meet PA recommendations, while those who reported long sleep duration and good sleep quality were more likely to follow PA recommendations [15]. Regular exercise has small-to-moderate beneficial effects on sleep duration while better sleep quality may be associated with more PA the next day [45]. Intervention in physical activity levels has been shown to improve sleep, but improved sleep did not result in increased physical activity levels. [46]. Moreover, a bi-directional effect between sleep and PA for sleep duration, but not for sleep quality [47]. Physical activity and sleep as factors that identify homogeneous groups of older adults may be of great practical importance, however, it is advisable to continue to clarify the relationship between these factors in further studies.

In the study group, the health-promoting diet index (pHDI) did not differentiate adherence to clusters which failed to verify the hypothesis that a more adequate diet characterized people who displayed a more health-promoting lifestyle. This result does not reflect existing knowledge about the relationship between diet and physical activity [15,48,49], and sleep duration [50,51]. An unbalanced diet and reduced physical activity were often reported in older adults [48]. The research showed that adherence to a vegetarian and modern pattern may be considered factors in maintaining good physical fitness, while a Western pattern may lead to poor physical fitness in older Chinese people [52]. The link between diet and physical activity is also confirmed by the identification of the ‘pro-healthy eating and more-active’ pattern in the Polish elderly [49]. The lack of a differential effect of pHDI on the clusters may be explained by more inconsistent results describing the relationship between diet and sleep duration, the more complex nature of the relationship [50], but also by more attention being previously paid to sleeping disorders [53] compared to sleep duration. Hence, in order to learn more about these connections in further research more attention should be paid to different sleep conditions, physical activity, and nutritional status of older adults [53]. Moreover, in profiling groups that are homogeneous in terms of lifestyle, but also in distinguishing them, other indicators describing eating behavior or nutritional status should also be included.

Socio-demographic characteristics such as age, gender, education, and place of residence were introduced into the cluster profiling, as their importance in determining the various lifestyle components of the elderly is confirmed by the results of available studies [54,55]. However, we did not note their significant impact on the adherence to the identified clusters. The lack of impact of either age or gender on the adherence to the ‘pro-healthy eating and more-active’ pattern was also found in another study in the Polish elderly [49]. This may support the rationale for identifying homogeneous groups on the basis of different lifestyle components in further research, rather than just looking for bilateral relationships. In contrast, self-reported financial status was found to be a predictor of adherence to the pro-healthy cluster. Those declaring “above average” financial status were less likely to belong to this cluster. This surprising result of the analyses may be caused by the specificity of this indicator. The study that used a more comprehensive indicator, incorporating financial status as one of its many components, namely socioeconomic status (SES), showed that a better socioeconomic status linked positively to the ‘pro-healthy eating and more-active’ pattern [49]. This is corroborated by previous studies that showed the positive impact of a higher socioeconomic status on attitudes toward healthy dietary patterns [56], dietary behaviors [57,58], dietary patterns [59], and nutritional status [60], as well as greater physical activity [61] in older adults. However, it should be noted that higher SES was associated with lower sleep duration [62] in older adults. Positive associations between diet, physical activity, and SES were also shown in the elderly population in Poland [56,57,63,64]. Thus, a higher socioeconomic status is generally associated with healthier choices [65,66], but this applies more to higher-income countries than others [67]. People with a higher SES in the latter group of countries may display more varied eating behavior [66,68], which may explain the result obtained in our study. Moreover, there are inconsistencies in the results regarding the relationship between physical activity and SES. Some studies suggest that higher SES is associated with lower levels of physical inactivity as well as higher levels of leisure-time PA [69,70], while others found the opposite results [71,72]. As there are some inconsistencies in the results regarding the relationship between dietary patterns, physical activity, and socioeconomic status, and rather single lifestyle elements are included in these considerations, further research with a more comprehensive approach is still required. It is also important to take into account both self-reported and objectively measured variables, as the inconsistencies in the results of the two methods occur. For example, self-reported PA showed negative associations with socio-economic status for both men and women, while objectively measured PA correlated positively with SES in men [55].

In the study group, the probability of adhering to the pro-healthy cluster increased with higher nutritional knowledge which supports the research hypothesis. In contrast, higher nutritional knowledge reduced the likelihood of adherence to the short sleep cluster. It should also be noted that although the low PA and short sleep clusters were characterized by similar levels of nutritional knowledge, the predictive effect of nutritional knowledge was observed only in the short sleep cluster. Increased nutritional knowledge in the pro-healthy cluster can be acquired during a nutritional education process, which usually includes nutritional recommendations and physical activity recommendations especially when the message is directed to people struggling with overweight and obesity [14]. In the NU-AGE study sample, it was confirmed that higher nutritional knowledge and nutrition-related attitudes scores were associated with higher physical activity, and also with lower BMI [56]. Positive associations between nutrition-related knowledge and physical activity were found in Belgian women [73]. The positive association between nutrition knowledge and healthy eating was confirmed in the previous studies [74,75], though the relationship is weak [75].

Self-reported health can be linked to lifestyle in various ways [76]. Although this study did not examine causal relationships, the predictive effect of self-perceived health was confirmed. Self-assessment of health as being the same or better than peers increased the chances of revealing a more pro-healthy lifestyle (an adherence to the pro-healthy cluster), and decreased the chances of adherence to the low PA cluster. In addition, a study by Jeruszka-Bielak [49] showed that older adults who assessed their health status as good or better in comparison with other people of the same age were more likely to adhere to the ‘pro-healthy eating and more-active’ pattern. A higher self-reported health may confirm better health and functioning, which may be due to the absence of symptoms associated with ageing, including muscle deterioration and other bothersome symptoms that lead to slower activity and a decline in wellbeing [77,78]. However, there is a lack of studies in which self-reported health is linked to objective health assessment, and then to lifestyle.

### Strengths and Limitations of the Study

The strength of the study is that the sample of elderly respondents was drawn from a representative sample of 1017 Polish adults. This was a national population-based sample, with a valid selection of the study group, carried out by professional interviewers. The stratification of the sample was based on the data on the demographics of Poland, published by the Central Statistical Office in Poland (CSO-GUS). However, only the subgroup of the elderly i.e., those over 60 years of age, were included in the analysis. Although the study group meets the characteristics of representativeness of the elderly it is specific to the Polish culture, which needs to be taken into account when considering other populations. There are also some other limitations regarding the study, which should be mentioned. The results presented here come from a cross-sectional study, which precluded assessment of the causal relationship between variables. The majority of variables were constructed using the self-reported data such as the financial status or health status, which may have been biased due to the subjective estimate. Despite the limitations demonstrated previously, our study provides an interesting insight into the inter-relationships of a large number of lifestyle components of older people and their associations with sociodemographic characteristics, as well as perceived health, nutritional knowledge, and quality of diet.

## 5. Conclusions

The results of the study confirmed the association between selected components of lifestyle in the elderly subgroup but only partially. Self-reported physical activity and sleep duration were found to be factors allowing to identification of homogeneous groups according to lifestyle while smoking cigarettes and frequency of alcohol consumption did not differ in these groups. Amongst the factors differentiating older people’s lifestyles, only self-reported financial status, self-reported health status, and nutritional knowledge determined cluster adherence. Gender, age, education, place of residence, BMI, and health-promoting diet index (pHDI) did not differentiate adherence to clusters. The likelihood of belonging to the pro-healthy cluster increased with nutritional knowledge and declaring the same or better health status in comparison with peers, while it decreased among the people who declared above-average financial status.

The obtained results importantly extend the previous findings by showing that the importance of individual elements of lifestyle should be also perceived in the context of other lifestyle-related factors. Indeed, not all bilateral relationships between lifestyle components were confirmed when a more comprehensive lifestyle approach was applied with the use of cluster analysis. The results of this study therefore encourage the continuation of research focused on lifestyle as a whole, which will be in line with the need to strengthen the implementation of multifaceted lifestyle interventions.

## Figures and Tables

**Table 1:** Characteristics of the study sample.

		Number of Respondents N = 271	%
Gender	Women	186	68.6
	Men	85	31.4
Age	60–65 years	64	23.6
	66–70 years	86	31.7
	71–75 years	51	18.9
	above 75 years	70	25.8
Education	Primary	87	32.1
	Vocational	99	36.5
	High school	72	26.6
	Higher	13	4.8
Place of residence	Rural area	120	44.2
	Town < 100 tys. Town > 100 tys.	98 53	36.2 19.6
Self-reported financial status	Below average (1 i 2)	38	14.0
	Average (3)	189	69.7
	Above average (4 i 5)	44	16.3
Self-reported health status	Worse than peers Same as peers Better than peers	43 169 59	15.9 62.3 21.8
BMI	Normal weight Overweight Obesity	26 170 75	9.6 62.7 27.7

**Table 2:** The identified clusters according to lifestyle characteristics.

	Total	Clusters			*p*-Value
		Pro-Healthy	Low PA	Short Sleep	
Percentage (number of respondents)	100.0 (271)	32.1 (87)	48.3 (131)	19.6 (53)	
Self-reported physical activity during the week *	1.5; 0.61	2.2 ^a^; 0.41	1.1 ^b^; 0.30	1.5 ^c^; 0.54	<0.001 *
Low (%, N)	54.6 (148)	1.1 (1)	90.1 (118)	54.7 (29)	<0.001 ****
Moderate (%, N)	39.1 (106)	80.5 (70)	9.9 (13)	43.4 (23)	
High (%, N)	6.3 (17)	18.4 (16)	0.0 (0)	1.9 (1)	
Self-reported physical * activity during weekends	1.5; 0.56	2.1 ^a^; 0.37	1.1 ^b^; 0.31	1.4 ^c^; 0.49	<0.001 *
Low (%, N)	56.5 (153)	3.5 (3)	89.3 (117)	62.3 (33)	<0.001 ****
Moderate (%, N)	40.2 (109)	86.2 (75)	10.7 (14)	37.7 (20)	
High (%, N)	3.3 (9)	10.3 (9)	0.0 (0)	0.0 (0)	
Sleep during the week **	1.9; 0.56	2.1 ^a^; 0.42	2.1 ^a^; 0.35	1.1 ^b^; 0.23	<0.001 **
Less than 7 h (%, N)	19.6 (53)	3.5 (3)	0.0 (0)	94.3 (50)	<0.001 ****
7–8 h (%, N)	68.6 (186)	81.6 (71)	85.5 (112)	5.7 (3)	
More than 8 h (%, N)	11.8 (32)	14.9 (13)	14.5 (19)	0.0 (0)	
Sleep during weekends **	2.0; 0.57	2.2 ^a^; 0.43	2.2 ^a^; 0.36	1.1 ^b^; 0.23	<0.001 **
Less than 7 h (%, N)	18.8 (51)	1.1 (1)	0.0 (0)	94.3 (50)	<0.001 ****
7–8 h (%, N)	67.2 (182)	78.2 (68)	84.7 (111)	5.7 (3)	
More than 8 h (%, N)	14.0 (38)	20.7 (18)	15.3 (20)	0.0 (0)	
Smoking cigarettes (%, N)	12.5 (34)	11.5 (10)	14.5 (19)	9.4 (5)	0.603 *
Frequency of drinking alcohol ***	1.8; 1.07	1.9; 1.04	1.8; 1.04	1.7; 1.21	0.128 ***

* Mean value from a three-point scale, where 1—low, 2—moderate, 3—high; *p*—significance between groups (Kruskall–Wallis H test). ** Mean value from a three-point scale, where 1—less than 7 h, 2—7–8 h, 3—more than 8 h; *p*—significance between groups (Kruskall–Wallis H test). ^a,b,c^—different letters indicate statistically significant differences; *p* < 0.05—Kruskal–Wallis H-test with adjustment by the Bonferroni correction. *** Mean value from a six-point scale, where 1—less than once a month or never; 2—1–3 times a month; 3—once a week; 4—a few times a week; 5—once a day; and 6—a few times a day; *p*—significance between groups (Kruskall–Wallis H test). **** significance between groups (Chi-square test)

**Table 3:** The characteristics of the identified clusters.

	Total % (N)	Clusters			*p*-Value
		Pro-Healthy	Low PA	Short Sleep	
Self-reported financial status					
Below average	14.0 (38)	16.1 (14)	15.3 (20)	7.5 (4)	0.015 *
Average	69.7 (189)	78.2 (68)	63.3 (83)	71.7 (38)	
Above average	16.3 (44)	5.7 (5)	21.4 (28)	20.8 (11)	
Self-reported health status					
Worse than peers	15.9 (43)	4.6 (4)	19.8 (26)	24.5 (13)	0.006 *
Same as peers	62.3 (169)	66.7 (58)	62.6 (82)	54.7 (29)	
Better than peers	21.8 (59)	28.7 (25)	17.6 (23)	20.8 (11)	
Nutritional knowledge (mean value; standard deviation)	12.5; 4.31	14.3 ^a^; 3.60	11.9 ^b^; 4.23	11.1 ^b^; 4.63	<0.001 **
Pro-healthy Dietary Index (pHDI) (mean value; standard deviation)	4.0; 1.75	4.1; 1.61	3.9; 1.76	4.0; 1.94	0.560 **
Body Mass Index (BMI) (mean value; standard deviation)	27.9; 4.19	27.7; 3.71	27.9; 4.48	28.2; 4.26	0.814 **

* Chi-square test, ** Kruskall-Wallis H-test, *p* < 0.05, ^a,b^—different letters indicate statistically significant differences; *p* < 0.05

**Table 4:** Cluster odds ratios (OR; 95% CI) in the study sample.

Variables	Pro-Healthy Cluster (Ref. Other Clusters)		Low PA Cluster (Ref. Other Clusters)		Short Sleep Cluster (Ref. Other Clusters)	
		*p*		*p*		*p*
Pro-healthy Dietary Index (pHDI)	1.05 (0.88–1.25)	0.548	0.94 (0.81–1.01)	0.403	1.05 (0.87–1.26)	0.591
Nutritional knowledge	1.18 (1.09–1.28)	<0.001	0.95 (0.89–1.00)	0.085	0.91 (0.85–0.98)	0.015
Financial status						
Average (ref. below average) Above average (ref. below average)	0.99 (0.46–2.16) 0.30 (0.09–1.00)	0.982 0.050	0.63 (0.30–1.29) 1.21 (0.48–3.02)	0.203 0.686	2.16 (0.71–6.62) 2.23 (0.62–8.06)	0.175 0.221
Self-reported health status						
Same as peers (ref. worse than peers) Better than peers (ref. worse than peers)	4.94 (1.61–15.15) 7.67 (2.31–25.57)	0.005 <0.001	0.63 (0.31–1.28) 0.39 (0.17–0.90)	0.202 0.028	0.55 (0.25–1.21) 0.58 (0.22–1.52)	0.136 0.270

*p*—the significance of the Wald’s test.

## Data Availability

Data presented in this study are available upon request from the corresponding author.
